# An exploratory qualitative study of the experiences of women in volunteer non-player roles in Australian community football clubs

**DOI:** 10.3389/fspor.2024.1448923

**Published:** 2024-12-04

**Authors:** Joanne M. Morgan, G. P. Lovell, K. Tulloch

**Affiliations:** ^1^ School of Health, University of the Sunshine Coast, Sippy Downs, QLD, Australia; ^2^ School of Psychology, University of Worcester, Worcester, United Kingdom

**Keywords:** community sport, thematic analysis, gender stereotypes, participation, barriers, facilitators, intersectionality, soccer

## Abstract

**Introduction:**

Women are underrepresented in volunteer non-player roles in community sporting clubs, particularly in traditionally male sports such as football (soccer), where participation rates for men and boys remain higher than women and girls. Experiences of women volunteering in community sporting clubs are not well-represented in research literature. By exploring women's experiences volunteering in community Australian Football clubs, the present research aimed to understand the barriers and facilitators of women's participation in volunteer non-player community sport club roles. Our intention is that our findings will provide empirical basis for the design of efficacious evidence-based interventions and initiatives to help close the gender gap of volunteerism rates and experiences, thus facilitating equal opportunities for women to access associated individual social, physical, and mental health benefits of sports volunteering.

**Method:**

We conducted individual semi-structured interviews with six women from four Australian Football clubs, to determine barriers and facilitators to volunteering.

**Results:**

Reflexive thematic analysis of barriers generated six themes: high expectations of self, intersectionality (of gender with motherhood or race), constrained resources, interpersonal disconnection, lack of organisational support and structure, and gender role assumptions and stereotypes. Analysis of facilitators produced six themes: having or building confidence, positive reinforcement, social connection, deliberate efforts to engage women, role autonomy and shaping, and supportive culture within a club or governing body.

**Discussion:**

Findings revealed that impacts on women's development in non-player roles exist at the individual level, including the interaction of gender, race, and parental status, but also extend beyond this to personal, interpersonal, organisational, and sociocultural factors. Given our research findings we make seven recommendations for governing bodies and community football clubs to enhance volunteering gender equity: 1. Establish support for women by dividing work evenly among volunteers; 2. Provide clear descriptions of non-player roles; 3. Match the skill sets of new women volunteers to suitable roles; 4. Implement mentorship programs to aid collaboration among women who volunteer; 5. Educate communities about gender biases and assumptions; 6. Monitor and seek feedback on gendered task allocation to ensure women's unpaid labour is not disproportionate to men's; and 7. Promote and publicise women in non-player roles to enhance women's visibility and acceptance in community football clubs

## 1 Introduction

Community sporting clubs are important to their local communities as they offer opportunities for physical activity involvement and interaction with others (1) while providing a strong sense of belonging and mutual support for club members, including non-player volunteers (2). Non-player roles are those positions responsible for essential tasks that enable sport to occur and include coach, official (e.g., referee), team manager, committee member, and other support roles. As community sporting clubs in Australia are predominantly non-profit organisations, they frequently rely on volunteers who freely elect to give their time for no monetary reward (3) fulfilling non-player roles (4).

Women are underrepresented in non-player roles in sport (5). In Australia, of the 5.9 million people who volunteer through an organisation or group annually, women are more likely to volunteer than men. Conversely, men are more likely than women to volunteer for sport and physical recreation organisations [47.5% and 30.5% respectively; (6)], especially in sports with higher participation rates in men and boys (5). For example, in football (soccer) in Australia, the context of this study, women account for only 32% of the 475,400 volunteers in non-player roles in community clubs (7). Further, there are gender differences in the types of roles undertaken with coaches, officials, and committee members more likely to be men, and team managers more likely to be women (8).

By exploring the experiences of women volunteering in community football clubs, the present research aims to understand the barriers and facilitators of women's participation in community sport volunteer roles. The purpose of this aim is that our findings will provide empirical basis for the efficacious design of evidence based interventions and initiatives that will help close the gender gap in terms of volunteerism rates and experiences between men and women. The intention is that our suggestions will not simply increase the already disproportionate unpaid labour load experienced by women. The intention, instead, is to enrich women's experiences of volunteering such that they can access the social, physical, and mental health benefits associated with volunteering in sport (9) in parity with men. Furthermore, through the suggestions based on our findings, this research will have impact not only upon women at a personal individual level, but also for sport and society.

The underrepresentation of women in non-player roles is a problem for sport, society, and women. Research shows that women in non-player roles also benefit by developing life skills and acting as a positive role model for other women and girls (10, 11). Individual benefits for women volunteers can include opportunities to build social networks, personal and career development, and increasing self-confidence (12–14). While this does rely on additional unpaid labour from women who may already hold a significant load, there are systemic benefits for women collectively that arise from women's volunteer efforts (15). Role models, such as women coaches, can strengthen girls' sense of inclusion in sports environments and enhance their confidence and self-efficacy (16, 17). The lack of representation of women in non-player roles at the community level introduces a negatively reinforcing cycle in which most participants are not exposed to women in non-player roles, which in turn is a barrier to women undertaking or remaining in non-player roles (18). This cycle constitutes a substantial limitation given the benefits of women role models in sport and can reinforce the negative perception that sport should be a predominantly men and boys' endeavour (19). Gender equity in non-player roles in community sport is crucial as it is often in this context that young people's sporting experiences begin, and often continue (20). Therefore, greater visibility of women in these roles can have profound impacts upon sporting communities and broader society by disrupting traditional institutional norms and challenging stereotypes relating to the suitability of men and women for particular roles (18, 21).

LaVoi's (22) ecological-intersectional model is a useful theoretical framework to examine and understand the multifaceted challenge of women's underrepresentation and experiences in sporting non-player volunteer roles along with the significant impact potential of women's involvement for the complex social issues at play, such as gendered power relations and gender role stereotypes, to move beyond tokenism. LaVoi's model – based around a feminist approach, intersectionality, and power – asserts that women coach development is complex, is influenced by various factors with powerful effects, positions intersectionality at the heart of the model, and can be conceptualised as being affected at four key levels. These four levels extend from micro to macro perspectives: (1) the individual level, involving factors such as self-perceptions, experience, and burnout; (2) the interpersonal level, encompassing social-relational influences such as relationships, colleagues, and friends, and immediate settings such as home, school or workplace; (3) the organisational level involving organisational practices, job descriptions, issues of discrimination, and opportunities (or lack thereof); and (4) the socio-cultural level, incorporating norms and cultural systems that indirectly influence women coaches. Intersectionality emphasises that a person's identities, such as gender, age, race, sexuality, social class, and ability interact on multiple levels with various forms of prejudice and contribute to systemic social inequality (23). Intersectional identities influence women and girls in non-player roles in sport, such as the intersection of gender and motherhood (10). Power was integrated at each level of LaVoi's (22) model and is bidirectional, indicating that power permeates all facets of social life from both top-down and bottom-up perspectives. Researchers draw on multiple conceptualisations to define power (24), such as hegemonic masculinity (25), which refers to the most dominant, socially-valued and socially-defined form of masculinity. In modern Western societies, this masculinity typically includes being strong, competent, and rational. Although comprehensive and well-established in the research of women coaches [e.g., (26, 27)], and recently used to explore barriers faced by women referees and officials in basketball (28), there is limited research utilising the ecological-intersectional model (22) across other non-player roles, and in community sporting contexts, such as football (soccer) clubs. Non-player roles in football clubs include coaches and officials, and other roles that are similar in that they have been traditionally held by men thus women in these roles may experience the same barriers at the individual, interpersonal, organisational, and socio-cultural levels. The present research provides a novel opportunity to explore women's non-player roles in Australian community football clubs from the ecological-intersectional lens, thus extending LaVoi's ecological intersectional model in the broader community sport volunteer context. The novelty of this research is that it extends the understanding of women's experiences in volunteer non-player roles beyond coaching and officiating roles [e.g., (27, 28)], contributing to the current body of knowledge to facilitate more equitable job crafting for women volunteers in sporting organisations.

Research examining the underrepresentation of women in non-player roles is evident in other non-voluntary areas of the sport labour force including governance, leadership and management (29–31). While the need for increased representation and inclusion of women in non-player roles has been recognised (29, 32), research on women's experiences volunteering in these roles in community sports organisations is scarce. Existing research suggests the representation of women in non-player roles in sport broadly is impacted by factors including dynamics of gender and power, role conflict, and available supports (27, 33). Sport is an important social institution, particularly in Australia, evidenced by its high participation rates and cultural dominance (34). Sporting club settings are commonly built on traditionally masculine values, such as toughness and competitiveness (35), thus gender and power dynamics influence women's involvement in non-player roles (18, 27, 36). Because non-playing roles in Australia were historically dominated by men (37), sporting volunteer contexts often reproduce traditional gender roles. Roles women undertake in clubs often replicate domestic roles, such as running the canteen and arranging uniforms (36). Women are regularly overlooked for sports coaching, officiating, and leadership roles due to presumptions they do not possess the required qualities such as toughness, competitiveness, and loudness that men do (38). Consequently, gendered discourses are perpetuated. Even when women undertake roles traditionally held by men, they often face gender stereotype challenges. For example, Tingle, Warner, and Sartore-Baldwin (18) interviewed eight former women basketball officials from American high school and college levels who concluded repetitive, low-intensity, disrespectful behaviours from male peers and supervisors led to their withdrawal from officiating. They concluded the women officials threatened the hegemonic masculinity and were therefore often overlooked for appointments based on gender, not ability. Hogan, Bowles, and Kitching (27) found women coaches from community Gaelic football clubs reported feeling like outsiders and experiencing gender and age-related bias after being treated differently by older men coaches. These studies illustrate some challenges women face in sport that are attributable to power and gender dynamics, and further highlight the need for systematic, structural changes to dismantle entrenched sexism.

While there are benefits to volunteering, gender impacts women's volunteering experience both directly, in the volunteering context, and indirectly, through their circumstances more broadly. A significant barrier to women's volunteering capacity in sport has been identified as the balancing of multiple social roles (10, 33). Research has identified that women in Australia perform 4.13 h of unpaid labour per day compared to men's 2.14 h (39) and this inequity has been linked to constraints in pursuing additional sport activities (40). Leberman and Lavoi (10) proposed a work-family-volunteer role triad in their qualitative exploration of lived experiences of eight mothers who coached their children in youth soccer in America. The study focused on how mothers negotiated multiple roles, as workers outside the home, mothers, and volunteer coaches. All women reported experiencing pressures between these roles and at times felt guilty, although women reported feeling they needed to justify their coaching involvement to themselves, and that their passion for sport provided this justification. Similarly, Sotiriadou and de Haan (33) reported that among women members of sports governing bodies there was a strong sense that it is harder for women with young children to juggle their roles. As such, the multiple social roles that women fulfil, in multiple contexts, may deter them from taking on a “third shift” (e.g., volunteer) in the role-triad – however, this is also under-examined in sports research.

Research has identified support that can increase the acceptance of women in sports and create a culture that values gender diversity (26, 33). Sotiriadou and de Haan (33) interviewed 18 men and women on triathlon boards and found that male equity champions (men who play a central part in supporting the inclusion of women and ensuring their viewpoints on gender equity are expressed) challenged existing stereotypes and introduced organisational changes that encouraged women to engage in leadership roles. This championing from men allowed women to feel valued in sports leadership roles and endorsed acceptance of women in sports organisations. Another study showed mentorship programs can also support women's development in non-player roles. Banwell, Kerr and Stirling (26) interviewed seven women from several different sports in Canada, following their participation in a 12-month women's coaching mentorship program. Findings suggested mentorship is beneficial on a personal and interpersonal level, however, has little influence on organisational practices and cultural norms impacting women in sport. As such, support has a positive influence on women in non-player roles, however, little is known about supports for community sport volunteers.

Overall, there is limited research directly examining experiences of women in volunteer non-player roles in community sport. Much of the recent literature is focused on women in paid roles or at intercollegiate and professional sports levels [e.g., (18, 33)]. Non-player roles in professional settings differ from those at a community level, as individuals in community sporting clubs are typically volunteering their time for no monetary reward. Furthermore, most studies focused on coach roles, and not on other non-player roles such as team manager, club president, or secretary. Additionally, many studies investigated organisational conditions in North America, which has cultural differences to Australian community sport organisations. These limitations necessitate further research specific to volunteer non-player roles in community sporting clubs in Australia.

Given women sporting volunteers' experiences are poorly represented in research literature, this project explored the involvement and experiences of women volunteers in Australian community football (soccer) clubs, to determine barriers and facilitators to volunteering in non-player roles. The context of football was selected as it has the most volunteers of all sports in Australia [467,000; (8)] and is heavily associated with masculinity (36) yet played by increasing numbers of women and girls (7). Based on the importance of this topic area and identified gaps in the current body of knowledge, the specific research question for this investigation was, what are the barriers and facilitators to women volunteering in non-player roles in community football clubs?

## 2 Materials and methods

### 2.1 Research design overview

A qualitative methodology with a social constructivist approach was employed in recognition that human development is socially situated, and knowledge is jointly created through interaction with others. Given the social nature of volunteering in community sporting clubs, this approach was considered appropriate to explore women's experiences in non-player roles. Data were collected using semi-structured interviews and analysed using Braun and Clarke's (41, 42) method of reflexive thematic analysis.

### 2.2 Researcher description

During this study the lead author JM, a white, female, feminist, Australian woman, was concurrently undertaking a volunteer non-player role in a community football club. She shares characteristics such as gender, age group, and motherhood with some participants, and experiences such as volunteering in the same southeast Queensland football region. Given her background, knowledge, and experiences, she adopted a constructivist standpoint and acknowledges how her identities and personal experience in sporting clubs undoubtedly influenced the questions posed and the stories presented. During her 18 months of experience as a volunteer on a community sporting club committee, she has witnessed some of the barriers and facilitators faced by women volunteering in non-player roles in community football clubs. This lived experience enhanced data collection and analysis by serving as a resource to build rapport and empathise with the participants' experiences, and produce knowledge (42).

Prior to research, author JM had met several participants through her volunteer role at a community football club. Interactions occurred at area meetings conducted by a football governing body, and at JM's club committee meetings or football games. As meaning and knowledge are recognised as contextual in reflexive thematic analysis (42), JM's subjectivity following these prior interactions helped to shape knowledge rather than jeopardise credibility.

The co-authors, KT and GL, also have experience in community sporting organisations. KT is a white, middle-aged cisgendered feminist Australian woman. She has previously volunteered for a community cycling group of which she was a participant, and which comprised mostly male cyclists. Her research work includes adults' connections with others. GL is a white heterosexual married male with dual Australian and UK citizenship. GL has children in sport and extensive experience of volunteering in sport as well working in sport as a professional in non-player role.

### 2.3 Study participants

Following institutional ethical approval from University of the Sunshine Coast Human Research Ethics Committee (S221726), participants were recruited June-August 2022 using several strategies. Football Queensland, the state governing body for the sport, emailed a recruitment flyer to all community clubs in one of their southeast regions. Participants were also recruited directly via phone or email contact details obtained from community football club websites, and through author JM's non-player volunteer networks.

Participation in this research was voluntary, with no participation incentives, and all participants provided informed consent. Homogenous purposive sampling (43) was used; eligible participants were aged over 18 years, identified as women, actively volunteered in a non-player role at a community football club within a southeast Queensland regional area, and had done so for a minimum of six months. Exclusion criteria stipulated participants must not have been in a paid role in a community sporting club or professional sports association.

Participants were six volunteers in non-player roles at one of four community football clubs, within a southeast Queensland regional area. All participants were mothers aged 36–57 years (*M* = 44.7, *SD* = 8.6), and working in paid employment concurrent to their volunteer role and parenting responsibilities. Five women had at least one child playing at their club, and one woman was a former player at her club. Volunteer non-player roles undertaken by participants included treasurer (*n* = 2), secretary (*n* = 2), coach (*n* = 1), and female football director (*n* = 1). Time in role ranged from 7 months to 11 years (*M* = 3.9 years, *SD* = 3.7). Specific participant details can be found in Table 1.

**Table 1:** Participant details.

	Age in years	Connection to club	Volunteer role(s) undertaken for club	Time in role(s)
Volunteer 1	37	Former club player	Executive title holder for women's participation	2 years
Volunteer 2	36	Two children play at club	Committee title holder	3 years
Volunteer 3	53	Two children play at club	Committee title holder	4 years
Volunteer 4	43	Two children play at club	Committee title holder	4 years
Volunteer 5	42	One child playing at club	Coach	7 months
Volunteer 6	57	One child playing at club	Committee title holder	11 years

For participant anonymity given the relatively few women holding identifiable roles in the region, specific roles have not been provided. “Executive title holder” refers to chief executive/operations/financial officers, directors and board members. “Committee title holders” refers to club presidents, vice-presidents, treasurers and secretaries.

### 2.4 Procedure

Data were collected from individual interviews with participants regarding their involvement and experiences of volunteering in their community football club. The interview process was semi-structured to allow for flexibility of responses to open-ended questions, and for the researcher to probe these responses, thereby yielding richer data (44). The focus of the questions was as follows: (1) reason for volunteering (What led you to start volunteering? How did you come to be involved as a volunteer?); (2) preconceptions (Did you have any reservations about volunteering before you started? What did you see as the positives and negatives of volunteering?); (3) reality (What do you now see as the benefits and challenges of volunteering? Do you consider that these challenges differ from those experienced by men volunteers? Do the challenges differ from your expectations?); and (4) women volunteer development (What would you have liked to have known before volunteering? What could have helped you?). Interviews were conducted in person over a three-month period*.* Interviews were recorded and professionally transcribed verbatim. Field notes were written during the research process to document what the digital voice recorder may not capture, such as emphases placed on certain issues and words, or any facial reactions or emotions displayed. Transcripts of audio recordings were reviewed and edited for accuracy then uploaded to NVivo qualitative data analysis software (45) for analysis**.**

### 2.5 Analysis

Given the exploratory nature of the research question, an inductive approach was utilised (46). Two separate thematic analyses were undertaken, one to determine barriers and one to determine facilitators. The first transcript was inductively coded into discrete nodes jointly by authors JM and KT, with the remaining transcripts coded by JM. The initial nodes were then reviewed, and duplications were removed before being divided into barriers or facilitators. Reflexive thematic analyses were conducted on these two separate categories, following methods outlined by Braun and Clarke (41, 42), which were reviewed by KT and adjusted with discussion and agreement.

Methodological integrity was maintained through the procedures of fidelity (how closely the method was followed) to the subject matter and utility in achieving research goals (47, 48). Fidelity was increased through data adequacy, achieved by interviewing women in non-player volunteer roles as they can shed light on the barriers and facilitators faced by women in community football clubs through lived experience; through recognition of the influence of JM's own perspectives and appropriately limiting that influence during interviews; and through inclusion of a balance of interpretive commentary and supporting quotes, to ensure findings are grounded in the data (47). Utility was maximised by considering and reporting findings that are insightful and meaningful given the dearth of literature on women volunteers in community sporting clubs, and by the research question being adequately addressed by the method and design of the study (47).

## 3 Results

Initial coding produced 63 nodes. These were reviewed for relevance and overlap, with four nodes removed at this stage. Nodes were then separated with 21 nodes identified as barriers to women volunteering in non-player roles in community sporting clubs, and 38 nodes identified as facilitators; these were thematically analysed separately. The thematic analysis of barriers generated six themes: gender role assumptions and stereotypes; lack of organisational support and structure; constrained resources; high expectations of self; intersectionality (of gender with motherhood or race); and interpersonal disconnection. Six themes were produced from thematic analysis of the facilitators: supportive culture within a club or governing body; deliberate efforts to engage women; role autonomy and shaping; having or building confidence; social connection; and positive reinforcement. Table 2 shows themes and sample quotes for barriers and facilitators.

**Table 2:** Themes and sample quotes regarding the barriers and facilitators to women in non-player volunteer roles.

Themes	Illustrative quotes
Barriers	
Gender role assumptions and stereotypes	“I think one of the downfalls of being female is that you do get roped into all those extra things that aren't being done… you know, like the cleaning, and the canteen as well.” (P2)
Lack of organisational support and structure	“I feel like volunteer organisations aren't always as supported with structure as they should be. It would be nice if there was some support, in our case, a Football Queensland or a Football Australia, or someone else who can provide solid administrative toolkits for a club. I think you'd find then more women can meaningfully contribute without baulking.” (P1) “No, we had to find our feet ourselves - self-taught.” (P3)
Constrained resources	“Time would be the biggest barrier. The biggest barrier. Without question.” (P1) “Challenges are the lack of volunteers, so it puts a strain on the committee.” (P4)
High expectations of self	“I knew it would be my demise [taking on a senior committee role]. You know, it would be a total set up for failure moment and which I wouldn't be okay with. I, I would put myself under pressure before I would give up.” (P1)
Intersectionality	“I think it's hard because of the time that's involved. Especially for single parents, to find extra time on top of bringing their kids to games, it would be a struggle.” (P4) “I also had reservations because I didn't know if I could do it, because I hadn't done it before in Australia.” (P5)
Interpersonal disconnection	“I don't have the day to day or hands on approach with the club. I'm not down there on a Saturday. I'm not getting to the games. While other people are really, really active, really, really present and understand the day-to-day stuff. I think if you can't be as present, it does take longer to understand operational stuff.” (P1)
Facilitators	
Supportive culture within club and governing body	“I suppose I’m a woman in a man's game, but I’ve never felt or been made to feel that way at all. I’ve always been welcomed, and I think I’ve always felt respected by the males in our game, be it players, be it club members, be it committee members, be it life members. I’ve never felt different because I’m a woman.” (P6)
Deliberate efforts to engage women	“A phone call from the president…. It was a call from, from the club's president, to say the club was really wanting to invest in women, in female football.” (P1)
Role autonomy and shaping	“Basically it became more of a conversation of, “what is the role about? What are we trying to achieve?”, and less about how you achieve it. So, I kind of was given a little bit of freedom….generating support that demonstrates it can be done differently.” (P1)
Social connection	“Positives definitely was that it was a very good club community. Very supportive, inclusive and yeah you could see that those positions became friendships as well as volunteer roles.” (P3)
Positive reinforcement	“When I talk to parents, they acknowledge that without me checking in and stuff, they probably wouldn't be at the club. That's always nice.” (P4)
Having or building confidence	“I had no reservations at all [about volunteering at a football club].” (P6)

### 3.1 Barriers

#### 3.1.1 Gender role assumptions and stereotypes

It is evident from the women interviewed that gender has influenced their experiences in non-player roles, particularly relating to gender stereotypes with one volunteer reporting:

I think sometimes, there's that perception around male dominated sport, you know, “what would you know, you’re a female?”, type of thought process. I guess it's that understanding of the game. That would be one of the stereotypes around it. (Volunteer 3)

Traditional male practices or an “old boys” club' were identified as a barrier as they can cause feelings of intimidation, with one volunteer explaining:

We would have our meetings at the tavern [an Australian tavern is a pub or bar]. It doesn't bother me, but I know for a lot of women it would be a real put-off to be going and sitting there by yourself with men that all played football. They all play as well, that's the other thing, whereas I had no idea, I didn't even know the rules of the game properly when I started (Volunteer 2).

The “old boys” club' also reportedly influenced the level of support received, with one women volunteer experiencing less support compared to peers who are men:

If you take a look at the teams in the age group I coach. The male coach has other males stepping up to help out. Whereas I haven't had any other male coaches or an assistant coach or parents step up to help (Volunteer 5).

Men and women adhering to traditional gender roles in clubs formed a barrier to women taking on roles typically undertaken by men. A volunteer, for example, reported:

It tends to be that the men have more structured positions of volunteering. The women still hold very traditional roles of volunteering – secretary, treasurer, canteen, kits, registrar…those types of things. You do still tend to see men in the higher and more authoritative positions and women more in the administrative (Volunteer 1).

#### 3.1.2 Lack of organisational support and structure

Several women conveyed that the lack of support from governing bodies, in providing structure for volunteers, was a barrier. For example, a volunteer stated, “*there was no real job description or position description given either, and I know they [Governing Body] don't actually have those either to give out, because I have enquired*” (Volunteer 2). The absence of a structured role description prevented women from volunteering and led to burnout due to no clearly defined parameters of the volunteer role, “*I think people get nervous about not knowing what's required of them*” (Volunteer 3), and “*If you try and do everything it just doesn't work then you burn out. I’ve definitely experienced volunteer burnout*” (Volunteer 2).

Most volunteers reported a lack of training from their club when beginning their volunteer role, resulting in more time being required than expected because they had to work out how to do tasks and source help themselves. One woman stated, “*I don't think I quite realised how much was gonna be involved. Like I thought there’d be some sort of a handover, which there wasn't. So, I basically had to figure everything out from scratch…*” (Volunteer 2).

#### 3.1.3 Constrained resources

Time constraints were identified as a barrier by most of the volunteers. For example, volunteers reported, “*I had reservations about my ability to commit the hours, the time more than anything*” (Volunteer 1), and “*I was spending a lot of time each week doing things for the club and then just got to the point where I was like, I can't keep doing this*” (Volunteer 2). A reported lack of volunteers exacerbates time constraints as more work is required from a small number of volunteers:

I'm extremely time poor. When you work 35 h a week and are probably still doing 20 h a week for the club, more at times – there's not enough hours in the day, and unfortunately there’s not the same amount of volunteers coming through the door as there used to be (Volunteer 6).

Another volunteer stated, “the negatives are just that you see the same faces doing all the work all the time” (Volunteer 3).

#### 3.1.4 High expectations of self

Volunteers' own high expectations contributed to role overload, with some women reporting:

For me personally, I can't leave things so if I know it's gotta be done and I can see it needs to be done but no one else is doing it, I’ll just do it. And so yeah, it was taking up a lot of my time (Volunteer 2).

Another stated, “That's me personally because if I give to a position then I like to give as much as I can, rather than doing a half effort job” (Volunteer 3). A self-expectation that one must have a rich understanding of a role to be able to volunteer in it prevented them from taking up that role. For example, one volunteer stated, “I love soccer, but I don't think I understand the game well enough to be able to implement good coaching sessions” (Volunteer 3).

#### 3.1.5 Intersectionality

Participants identified additional roles or identities that may have interacted with forms of prejudice to affect their experience as a woman volunteer in a community football club. The intersection of gender and motherhood was a challenge with most participants conveying difficulties in juggling multiple roles:

My level of involvement has to be managed around kids and work and everything else. Irrespective of what era we are in, women are still balancing a lot in any given day. And typically, it’s disproportionate. There's usually a dominant role that women have to play in the house, in the family. Not have to but do (Volunteer 1).

It was noted by one woman that this was a distinct difference between men and women volunteers:

If one of the men who'd played at the club, in the same situation as me, had played at the club and continue to be involved..I'm not sure that they would have to contemplate the balance of priorities the same way (Volunteer 1).

Being from a different race and culture was also identified as a barrier:

I think a lot of kids have trouble understanding my accent. So, there's some cultural differences, not a lot, but I obviously speak differently and some of the words that I use are not the same words that are used here (Volunteer 5).

#### 3.1.6 Interpersonal disconnection

Women reported having reservations about volunteering due to not knowing others on the club committee with one stating, “*Only working with people that I didn*’*t know…You work quite closely with the president and other committee members and what if you don*’*t get along, that could make it hard*” (Volunteer 4).

### 3.2 Facilitators

#### 3.2.1 Supportive culture within club and governing body

Several volunteers identified that set role descriptions from the club or governing body would facilitate more women volunteering. For example, one woman stated:

I think to attract more females, maybe having those set role descriptions so they know what it is. I think maybe females would feel more keen to say yes to the role if they knew they just had to stick to that, and they weren't going to be forced into doing a bunch of other things (Volunteer 2).

Support from a club in accepting the level of service a volunteer could contribute was a facilitator experienced by one woman:

Being able to be really honest about what I could do..And then, having that acknowledged as okay. There was no, “Oh, well then, you're not the right person because you're not available enough.” It was, “no, this is what we need, and you don't have to be there [for more time than you can provide]” (Volunteer 1).

#### 3.2.2 Deliberate efforts to engage women

Being personally invited to a volunteer role, or encouraged to become involved was a facilitator for successfully recruiting women:

The leaders are always open to new ideas and they don't stereotype against women. They do try and empower you and say, “yes you can do this role”, or “would you be interested in this role?”, so that builds confidence in your abilities which is nice. (Volunteer 3).

#### 3.2.3 Role autonomy and shaping

The ability for women to shape a volunteer role to suit one's individual skills and time availability was a reported facilitator:

I think if there was consideration for skill-based appointment, rather than time-based availability, it might be helpful. Because it might be that, you know, the skills of women and particularly balancing, and prioritizing, and targeting and focusing. Like being able to say, I can take that specific function and do it well. But I can't do that if you need me at the club 12 h a week running a bar. If what you're asking me to do is to provide strategic direction… and not be down here in a yellow vest as a ground official all weekend… I think I've got good skills for you (Volunteer 1)

#### 3.2.4 Social connection

All volunteers reported experiencing social benefits through volunteering at their club. One woman stated, “*Definitely meeting lots of new people, and this being such a new community, everyone's really keen to get to know one another. So yeah, I've met lots of great people through it and created friendships”* (Volunteer 2). It was reported that other women being involved at the club was a facilitator for attracting other women volunteers, “*I think definitely when you have women involved initially then that attracts more to come in. I found it was much nicer once another female was there”* (Volunteer 2).

#### 3.2.5 Positive reinforcement

Positive feedback from club members was reported as a facilitator for women volunteering. One woman affirmed:

I do get some good feedback from the girls [young girls who are club players]. The parents tell me the girls really like having a female coach, which is really nice. Growing up I never had a female coach, but I see how it would be a positive. The girls saying, “we love you as a coach”, “we hope you come back next year” is nice (Volunteer 5).

The acquisition of new skills is another reward mentioned by a volunteer, “Benefits are that it helped me with my skills…. Because there’s a variety of tasks, you’re upleveling in so many different areas which is great*”* (Volunteer 3).

#### 3.2.6 Having or building confidence

A level of self-confidence facilitated women taking on volunteer roles in football clubs. For example, a woman stated, “*I was totally comfortable [being the only female volunteer on the committee]”* (Volunteer 6). It was also reported that interventions that build confidence would facilitate more women getting involved in coaching:

There are mums that do help with coaching but they're not willing to step up into the coach role, so if we could provide more coaching sessions so they can actually build their confidence, instead of working under the male coach, I think that would be a really good thing. Particularly as a role model for our girls coming through (Volunteer 4).

## 4 Discussion

This study aimed to explore the involvement and experiences of women volunteers in community football clubs, to determine barriers and facilitators. This research addressed a gap in the literature by illuminating the lived experiences of women volunteering in various non-player roles at the grass-roots level of football, highlighting facilitators as well as barriers.

When discussing barriers, participants indicated that the role of a non-player volunteer is very demanding, requiring a high level of commitment, reliability, and time. Demand is often due to constrained resources such as a lack of volunteers, resulting in a large amount of work being distributed across a small number of people on the club committee. However, overload of a small number of volunteers may have also resulted from poor volunteer recruitment, strategy, and utilisation. These requirements placed stress on the women, particularly as all participants worked in paid employment and had family responsibilities concurrent to their volunteer role. In some cases, this stress is the result of participants' own high expectations of themselves in aspiring to do well in the role and in not wanting to let their club down or leave tasks for someone else. Many participants explained that they had to figure out how to perform the role themselves and emphasised that a lack of structure, in terms of no role descriptions or job training, contributed to them putting in more hours than expected prior to taking on the role. Consequently, some women reported experiencing burnout. It was not until specifically questioned about how the challenges women face might differ to those faced by peers who are men, that the women in this study identified any gender-based discrimination. In part, this may be due to the stigma of volunteering, whereby our participants may have been aware that it is difficult to attract and retain volunteers across a range of roles, interests and community groups (49); thus, this is not necessarily a social issue of gender but rather one of a perceived lack of time for many members of the community. Nevertheless, our participants acknowledged football is traditionally a male sport, with evidence their clubs reinforced this. Women reported occurrences of adhering to stereotypical women's roles, such as canteen or cleaning roles, and that men at the club avoided doing such tasks or left them to women. Participants also commented on experiences of feeling intimidated at the thought of having to speak to some men at the club, particularly those who had been there long-term. There was a perception that women were seen as less knowledgeable about the game.

Despite the women reporting several barriers, there was still an overall sense of enjoyment and satisfaction in volunteer roles, with various facilitators being described. Having confidence in oneself to undertake a non-player role and to fully commit to a non-player role and face any challenges that arise, especially when acknowledging that few, if any, other women were involved, were identified as facilitators. Acquiring new skills and receiving positive feedback from club members reinforced participants' enjoyment of their roles. There was an overwhelming sentiment that participants were highly motivated to contribute to the sport and/or community by volunteering. This motivation to contribute was inspired by their passion for football, desire to make improvements to how the club was being managed, aspiration to be a role model, or their need to give back to a sport that had given so much to them and their family. Many participants reported how social connections facilitated through their non-player roles, and the strong sense of belonging that they experienced created an emotional connection to the club which was positive. Where connections to the club existed prior to volunteering, for example playing at the club themselves or their children playing at the club, volunteers were more inclined to step into a non-player role.

A supportive club culture was a facilitator for participants. This support was provided through role training, through women being included in decision-making at their club, and through committee and club members being welcoming and respectful of women. Although our participants spoke explicitly in terms of their involvement being facilitated by men at their respective clubs, many participants stressed that availability of clear role descriptions and objectives would facilitate more women undertaking volunteer roles in community football. Where clubs had made deliberate efforts to engage women in non-player roles, women were more likely to undertake them. These efforts included intentionally recruiting women in non-player roles and building confidence in women by reassuring them that they are capable of such roles. A participant reported that a deliberate effort by her club to bring together stakeholders of female football within the club, through a message group and meetings, provided support and connection. In addition to these efforts, participants reported that clubs that supported women by allowing them to shape a non-player role to fit with other responsibilities, and the time and skills available, were facilitating the involvement of women. For example, women reported engaging in non-player roles in job-share arrangements and being allowed the autonomy to work from home and shape the role to suit their availability, rather than being required to be present at the club. Through women's increasing authentic involvement in sporting organisations at all levels, women can assert their own power as active participants, ensuring current barriers are not perpetuated.

Our current study was framed within LaVoi's (22) ecological-intersectional model, and while this model was developed specifically for researching women coaches, it has been appropriate here in examining the experiences of women in coaching and other non-player roles such as committee members (e.g., secretary, treasurer). Broadly, the themes that were identified in our research illustrate that barriers and facilitators to women volunteering in non-player roles in community football clubs exist at the individual level, including the interaction of gender, race, and parental status, but also extend beyond influences of the individual. Numerous interpersonal, organisational, and sociocultural factors were found to impact upon participants' development in their non-player roles. In sharing their stories, suggestions for clubs to address some of the barriers faced by women and enhance the facilitators mentioned have been established. Practical implications are integrated throughout each section.

The impact of intersectional roles and responsibilities were compounded for the women in this study, who were unaware of the time and exact tasks required of their role prior to taking it on and therefore highlighted the requirement for support and structure through clear role descriptions and objectives. This requirement for clear and structured role descriptions is consistent with Hogan, Bowles, and Kitching (27), who proposed that volunteer coaches at the community level would benefit from a starter pack which included a clearly defined role description and the requirements of new volunteers. This could be extended to other non-player roles in community sporting clubs. Women in our study reported that the ability to shape a non-player role to suit their individual skills and abilities was a benefit. It is therefore crucial that clubs understand their volunteers, so they can match their skill sets to suitable roles or identify opportunities for skill-matching, and development with other community members. In this regard, Egli, Schlesinger, and Nagel (50) asserted that coordinating the needs and expectations of volunteers is essential to their longevity and commitment to the role, and recommended the use of an entry questionnaire, to aid in meeting volunteers' expectations. Thus, it is essential that governing bodies and community football clubs establish support for women at the organisational level by dividing work evenly among volunteers, by providing a clear outline of the non-player role, for example through the provision of a starter pack, and by understanding new women volunteers to match their skill sets to suitable roles.

Support at the interpersonal level appears key for women volunteering in community football clubs. Similar to findings by Clarkson, Cox, and Thelwell (51), this study suggests that social connections and support networks are important facilitators of women volunteering in football contexts. For participants of the present study, a volunteer coordinator may have been able to capitalise on social connection (15), deliberate efforts to engage women, and supportive club environment, while preventing or minimising the impact of constrained resources, interpersonal disconnection, and lack of club support and structure. For example, to establish appropriate support structures for women volunteering in community sporting clubs, Hogan, Bowles, and Kitching (27) recommended a volunteer coordinator role at the administration level in clubs. This role was responsible for facilitating inductions for new volunteers, regularly checking in with volunteers and arranging collaboration among other women volunteers. As with a volunteer coordinator, mentorship could provide support at the interpersonal level. Many women in the current study aspired to be role models for other women and girls, and it was reported that having other women involved in non-player roles at their club was helpful, suggesting that mentoring is valuable. Mentoring was explored in a study by Banwell, Kerr, and Stirling (26) who examined the benefits of a mentorship program for women sporting coaches. Results showed that mentorship had a strong impact at the interpersonal level of the ecological intersectional model (22) with women developing connections with other coaches and individuals in sport. A mentorship program for women volunteering in non-player roles in community football clubs may have similar success and increase the number of women volunteering.

Support for women at the organisational level, from both the sporting club and the organising body appears important. Overwhelmingly the most common barrier for participants in the present study was time demands, due to a lack of volunteers at the club, which is consistent for volunteering roles more globally and beyond the sporting community [e.g., (49)]. This was consistent with the barrier of men at the club avoiding tasks stereotypically seen as women's work, which is further reflected societally and culturally in many countries, where tension exists in the joint responsibility for care and domestic tasks (52). The consequent lack of volunteers resulted in a large amount of work being distributed across a small number of people, which has been recognised as the workload of volunteers by Nagel et al. (53), who proposed community sporting clubs could recognise and support their volunteers via workload division. Similarly, Hogan, Bowles, and Kitching (27) asserted that distributing the workload and not demanding too much from a small number of volunteers is important for women coaches as they tend to have more responsibility for caring roles in the household – a societal fact that is recognised in many countries [e.g., (52)]. High organisational support has been recognised as a strong predictor of low volunteer turnover rates (49), emphasising its importance for diverse individuals in a range of settings.

While the societal level is the most distal from the individual, all women in this study had faced gender bias or recognised it exists. There is more to be done before women volunteering in football are accepted as the norm and not affected by such bias. Societal views need to shift to allow women to be considered equal to their peers who are men as typical social norms in sports roles often impede opportunities for women (54). Nevertheless, the objective is not to consider men as the opposition but to view them as allies who can work with women in community sporting clubs, increasing awareness of unconscious biases and stereotypes so they can be removed or at the very least reduced (27). Such framing has the potential to improve club culture and ensure Australian community football clubs are environments in which women are welcomed rather than received with hostility. Therefore, education regarding biases and assumptions as well as promoting and publicising women in non-player roles is necessary for women's visibility and acceptance in community football clubs. This will further need to extend to the structural systems of the clubs, including their policies and procedures, to ensure women's participation is not just accepted but actively sought and upheld as valuable. Initiatives to improve such participation will need to ensure appropriate alignment between proposed strategies and clubs' capacity to implement women's leadership training and positions (55).

A limitation of this study is the homogeneity of participants, and future research may consider how other identities of women volunteering in non-player roles intersect to produce different experiences in community sport, for example their sexuality, ethnicity or ability. A further limitation is that only women who had been volunteering for a minimum of 6 months were interviewed, so were seemingly having an overall positive experience in their club roles. Consequently, the views of women who may have been unable to overcome barriers were not heard. It is also possible that the views presented here were of more progressive clubs, as clubs with fewer women volunteers may reveal results and themes similar to ours, experienced at more intense levels than the volunteers in our study.

To address these limitations, future research could triangulate other stakeholders' perspectives within clubs, such as players, parents, members, and a greater number of diverse volunteers (e.g., age, carer status, cultural or linguistic background, from wealthier vs. disadvantaged clubs), to further identify and respond to women's participation barriers. This may even involve reflective practice with male club stakeholders to determine self-identified behaviours and attitudes that could be targeted within clubs. Changes to study designs that may facilitate this could include more open interviewing techniques for richer scope, or a more longitudinal design to investigate the relative success of proactive strategies for women's involvement. Furthermore, research should also consider how these barriers and facilitators presented in our research are specific to women volunteers. Finally, research must be conducted on the ways in which club systems, policies, procedures, and culture can be addressed to ensure community football clubs are psychologically safe for all individuals to contribute. Failing to address the structural workings of such organisations may mean limited success for increasing women's participation.

In conclusion, our interpretation of the findings, framed within the ecological-intersectional model (22), led us to some practical suggestions to support women volunteers in non-player community sport club roles: 1. Establish support for women by dividing work evenly among volunteers; 2. Providing clear descriptions of non-player roles; 3. Matchi the skill sets of new women volunteers to suitable roles; 4. Implement mentorship programs to aid collaboration among women who volunteer; 5., Educate communities about gender biases and assumptions; 6. Monitor and seeking feedback on gendered task allocation to ensure women's unpaid labour is not disproportionate to men's; and 7. Promote and publicise women in non-player roles to enhance women's visibility and acceptance in community football clubs. It is hoped that this research will resonate with stakeholders of community football and lead to increased support and development for women in volunteer non-player roles. This research has further illustrated the application of LaVoi's (22) ecological-intersectional model in the volunteer non-player context, expanding on previous use with female coaches. While this research included some limitations such as a small and homogeneous sample, consideration of these women's experiences in this exploratory study may provide insight for future research with varied methodologies and broader samples for triangulation. Additionally, the present research may aid the design of programs to support the development of women in non-player volunteer roles to increase gender equity in community sporting clubs, thus helping to facilitate equal opportunities for women to gain access to the associated individual social, physical, and mental health benefits of volunteer in sport.

## Data Availability

The datasets presented in this article are not readily available because only the research team will have access to transcripts. Requests to access the datasets should be directed to ktulloch@usc.edu.au.
